# Reducing Nav1.6 expression attenuates the pathogenesis of Alzheimer's disease by suppressing BACE1 transcription

**DOI:** 10.1111/acel.13593

**Published:** 2022-03-30

**Authors:** De‐Juan Yuan, Guang Yang, Wei Wu, Qi‐Fa Li, De‐en Xu, Michael Ntim, Chun‐Yan Jiang, Ji‐Chuan Liu, Yue Zhang, Ying‐Zi Wang, Dan‐Dan Zhu, Supratik Kundu, Ai‐Ping Li, Zhi‐Cheng Xiao, Quan‐Hong Ma, Shao Li

**Affiliations:** ^1^ Department of Physiology College of Basic Medical Sciences Liaoning Provincial Key Laboratory of Cerebral Diseases National‐Local Joint Engineering Research Center for Drug‐Research and Development (R&D) of Neurodegenerative Diseases Dalian Medical University Dalian China; ^2^ Department of Neurology and Clinical Research Center of Neurological Disease The Second Affiliated Hospital of Soochow University Suzhou China; ^3^ Jiangsu Key Laboratory of Neuropsychiatric Diseases Institute of Neuroscience Soochow University Suzhou China; ^4^ The Affiliated Wuxi No. 2 People’s Hospital of Nanjing Medical University Wuxi China; ^5^ Department of Thoracic Surgery Tongji Hospital Tongji Medical College Huazhong University of Science and Technology Wuhan China; ^6^ Development and Stem Cells Program Monash Biomedicine Discovery Institute and Department of Anatomy and Developmental Biology Monash University Melbourne Victoria Australia

**Keywords:** Alzheimer's disease, amyloid‐β, BACE1, hyperexcitability, Nav1.6 sodium channel, NFAT1

## Abstract

Aberrant increases in neuronal network excitability may contribute to cognitive deficits in Alzheimer's disease (AD). However, the mechanisms underlying hyperexcitability of neurons are not fully understood. Voltage‐gated sodium channels (VGSC or Nav), which are involved in the formation of excitable cell's action potential and can directly influence the excitability of neural networks, have been implicated in AD‐related abnormal neuronal hyperactivity and higher incidence of spontaneous non‐convulsive seizures. Here, we have shown that the reduction of VGSC α‐subunit Nav1.6 (by injecting adeno‐associated virus (AAV) with short hairpin RNA (shRNA) into the hippocampus) rescues cognitive impairments and attenuates synaptic deficits in APP/PS1 transgenic mice. Concurrently, amyloid plaques in the hippocampus and levels of soluble Aβ are significantly reduced. Interfering with Nav1.6 reduces the transcription level of β‐site APP‐cleaving enzyme 1 (BACE1), which is Aβ‐dependent. In the presence of Aβ oligomers, knockdown of Nav1.6 reduces intracellular calcium overload by suppressing reverse sodium–calcium exchange channel, consequently increasing inactive NFAT1 (the nuclear factor of activated T cells) levels and thus reducing BACE1 transcription. This mechanism leads to a reduction in the levels of Aβ in APP/PS1 transgenic mice, alleviates synaptic loss, improves learning and memory disorders in APP/PS1 mice after downregulating Nav1.6 in the hippocampus. Our study offers a new potential therapeutic strategy to counteract hippocampal hyperexcitability and subsequently rescue cognitive deficits in AD by selective blockade of Nav1.6 overexpression and/or hyperactivity.

## Graphical abstract

In the presence of Aβ oligomers, knockdown of Nav1.6 reduces intracellular calcium overload by suppressing reverse sodium‐calcium exchange channel, consequently increasing inactive NFAT1 (the nuclear factor of activated T cells) levels and thus reducing BACE1 transcription. This mechanism leads to a reduction in the levels of Aβ in APP/PS1 transgenic mice, alleviates synaptic loss, and improves learning and memory disorders in APP/PS1 mice after down‐regulating Nav1.6 in the hippocampus.

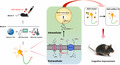

## 1 INTRODUCTION

Alzheimer's disease (AD) is a progressive, irreversible neurodegenerative disorder that pathologically characterizes senile plaque depositions in the brain composed of amyloid‐β (Aβ) produced by sequential cleavage of amyloid precursor protein (APP). As a key enzyme for APP cleavage, β‐site APP‐cleaving enzyme 1 (BACE1) is the key rate‐limitation enzyme in the process of Aβ production. Elevated levels of BACE1 have been observed in the brains of AD patients and model mice, with unclear mechanisms (Deng et al., 2016; Fukumoto et al., 2002; Yang et al., 2003). Inhibiting the expression or activity of BACE1 exhibits promising effects in attenuating cognitive deficits and other AD‐related pathology in both animal models and the early phases of clinical trials (Cummings et al., 2020; Deng et al., 2016; Hu et al., 2018; Peters et al., 2018). Thus, inhibition of BACE1 expression or activity is regarded as one of the prime strategies of AD therapy.

Hyperexcitability of neural networks is one of the early manifestations of AD (Haberman et al., 2017; Vico Varela et al., 2019) and plays an essential role in AD pathogenesis. Suppression of neural hyperexcitability, either with antiepileptic drugs or with the manipulation of inhibitory interneurons, attenuates cognitive deficits in AD model mice (Andrews‐Zwilling et al., 2010; Haberman et al., 2017; Lu et al., 2020; Sanchez et al., 2012; Tong et al., 2014; Zhang et al., 2014). Conversely, eliciting long‐lasting neuronal hyperexcitability accelerates the accumulation of Aβ deposition in the brains of APP transgenic mice (Yamamoto et al., 2015). Consistent with this observation, a study has found that lamotrigine, a nonselective sodium channel blocker, prevents neural hyperexcitability and reduces BACE1 levels and Aβ generation in APP/PS1 mice when administered chronically (Zhang et al., 2014). These studies highlight the causal contribution of neural hyperexcitability in Aβ accumulation. However, the mechanisms underlying these phenomena still remain unclear.

Voltage‐gated sodium channels (VGSC or Nav) are involved in the formation of action potentials of excitable cells and directly influences the excitability of neural networks. A VGSC consists of a single α‐subunit (220−260 kDa) with one or more small β‐subunits (30−40 kDa). The α‐subunit is the core protein of VGSC that contain nine isoforms (Nav1.1–Nav1.9), encoded by the SCN(X)A genes, distributed in different tissues. The main neuronal VGSC subtypes in central nervous system are Nav1.1, Nav1.2, and Nav1.6 (Abdelsayed & Sokolov, 2013). Nav1.1 is expressed predominantly in the interneurons (Frank et al., 2006; Ogiwara et al., 2007) and is primarily localized in the neuronal somata of GABAergic neurons (Ogiwara et al., 2007; Ragsdale, 2008; Yu et al., 2006). Cognitive deficits and working memory impairment have been associated with Nav1.1 alterations (Bender et al., 2016). However, Nav1.2 and Nav1.6 exhibit preference for excitatory pyramidal neurons. Nav1.2 is distributed in immature unmyelinated axons in the central nervous system during brain development (Ragsdale, 2008). In contrast, Nav1.6 being encoded by the gene SCN8A, clusters at the axon initial segments on the nodes of Ranvier in myelinated axons, where it generates action potentials facilitating saltatory conduction (Trimmer & Rhodes, 2004). Nav1.6 plays essential roles in neural excitability via modulating persistent current, resurgent current, and repetitive neuronal firing. Our previous studies reported that APP colocalized with Nav1.6 at nodes of Ranvier and axon initial segments (Xu et al., 2014). APP enhanced the Nav1.6 currents through a Go‐coupled JNK pathway (Li et al., 2016). The sodium current density of Nav1.6 is also enhanced by the presence of Aβ1‐42 oligomers (Wang et al., 2016). It also have been demonstrated that Nav1.6 is a crucial player in mediating the increase in spike frequency and hippocampal hyperexitability induced by Aβ1–42 (Ciccone et al., 2019). All these studies indicates the roles of Nav1.6 in AD pathogenesis. Thus, in the present study, we show that the expression of Nav1.6 is upregulated in the brains of APP/PS1 mice. Overexpression of Nav1.6 enhances BACE1 transcription through activation of the NFAT1 pathway in a way dependent on Na^+^ efflux and Ca^2+^ influx (via Na^+^/Ca^2+^ exchanger channel). Knockdown of Nav1.6 in the hippocampus attenuates the accumulation of Aβ and improves the synaptic plasticity and cognitive deficits in APP/PS1 transgenic mice. The present study reveals a novel mechanism of neural network hyperactivity in AD.

## 2 RESULTS

### 2.1 Elevated levels of Nav1.6 in the brains of male APP/PS1 mice with aging

**FIGURE 1:**
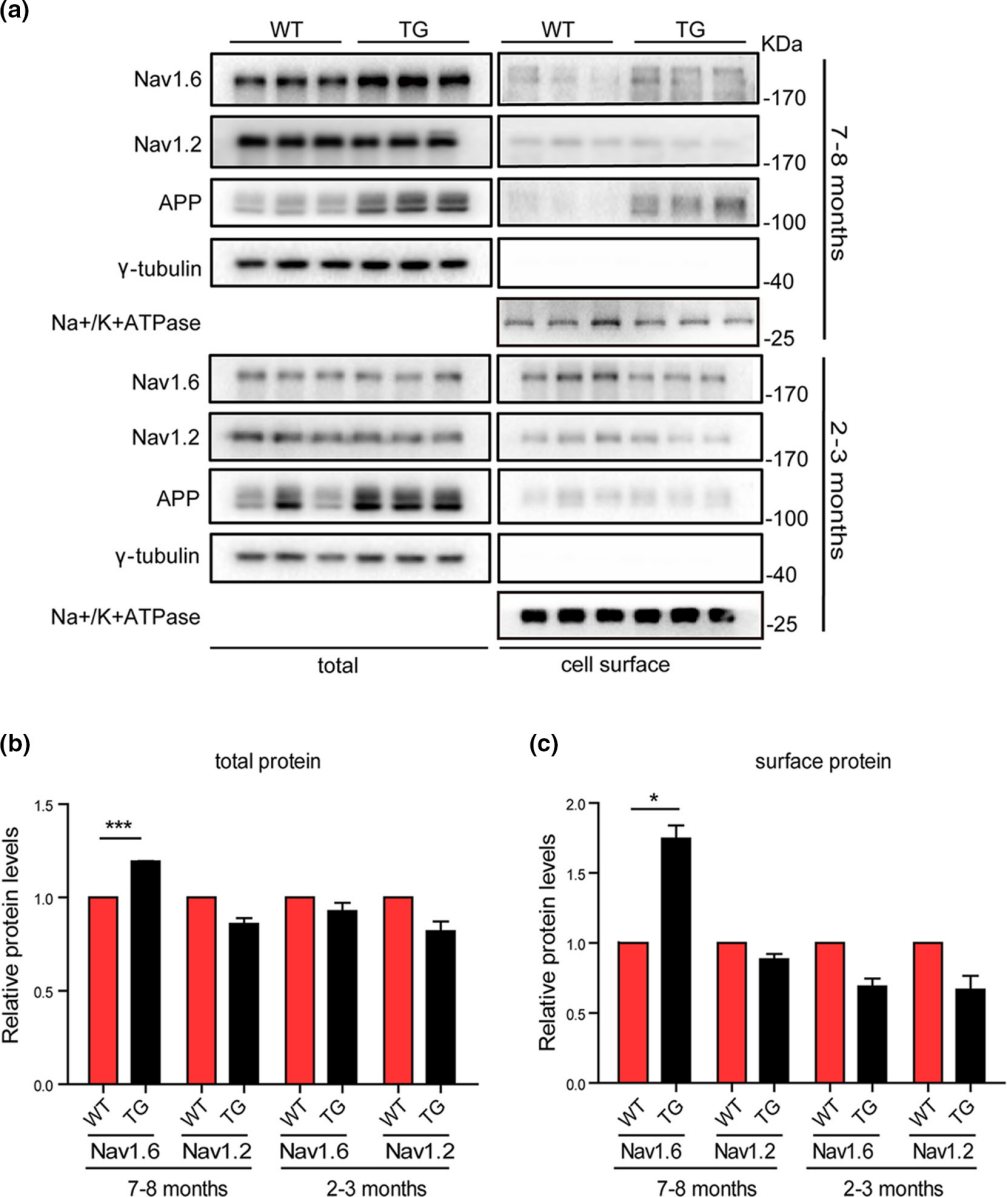
Expression levels of total and cell surface levels of Nav1.6/Nav1.2/APP in APP/PS1 mice brain. Representative immunoblots (a) and densitometry analysis of the total (b) and cell surface (c) protein expression levels of Nav1.6, Nav1.2. APP levels in the brain of APP/PS1 and WT mice at different ages (2–3 months and 7–8 months). Here, Nav1.6, Nav1.2, APP, and their corresponding γ‐tubulin immunoblots was performed on different parts of the PVDF membrane of different gels. Data were presented as mean ± SEM, *p < 0.05, and ***p < 0.001

To explore whether Nav1.6 participates in the pathogenesis of AD, we measured the expression levels of Nav1.6 in whole cell and cell surface of the brains of APP/PS1 mice separately at different developing ages. The results showed that these transgenic mice exhibited no AD‐like pathology, and displayed comparable levels of Nav1.6 both in the whole cell and the cell surface at 2–3 months of age (Figure 1), whereas significant elevated levels of Nav1.6 in the whole cell fractions and the cell surface were observed in the brains of APP/PS1 mice compared to the WT mice at 7–8 months of age, the time when APP/PS1 mice harbored extensive Aβ plaques and showed cognitive deficits (Zhang et al., 2014). Interestingly, the level of Nav1.2, another subtype of voltage‐dependent sodium channels, which usually distributes to unmyelinated axons at the developmental stage, exhibited no significant difference between APP/PS1 transgenic and WT mice (Figure 1). These results elucidated the link between Nav1.6 and AD pathogenesis.

### 2.2 Knockdown of Nav1.6 attenuates cognitive deficits, ameliorates suppressed synaptic plasticity, and reduces hyperexcitability in APP/PS1 mice

**FIGURE 2:**
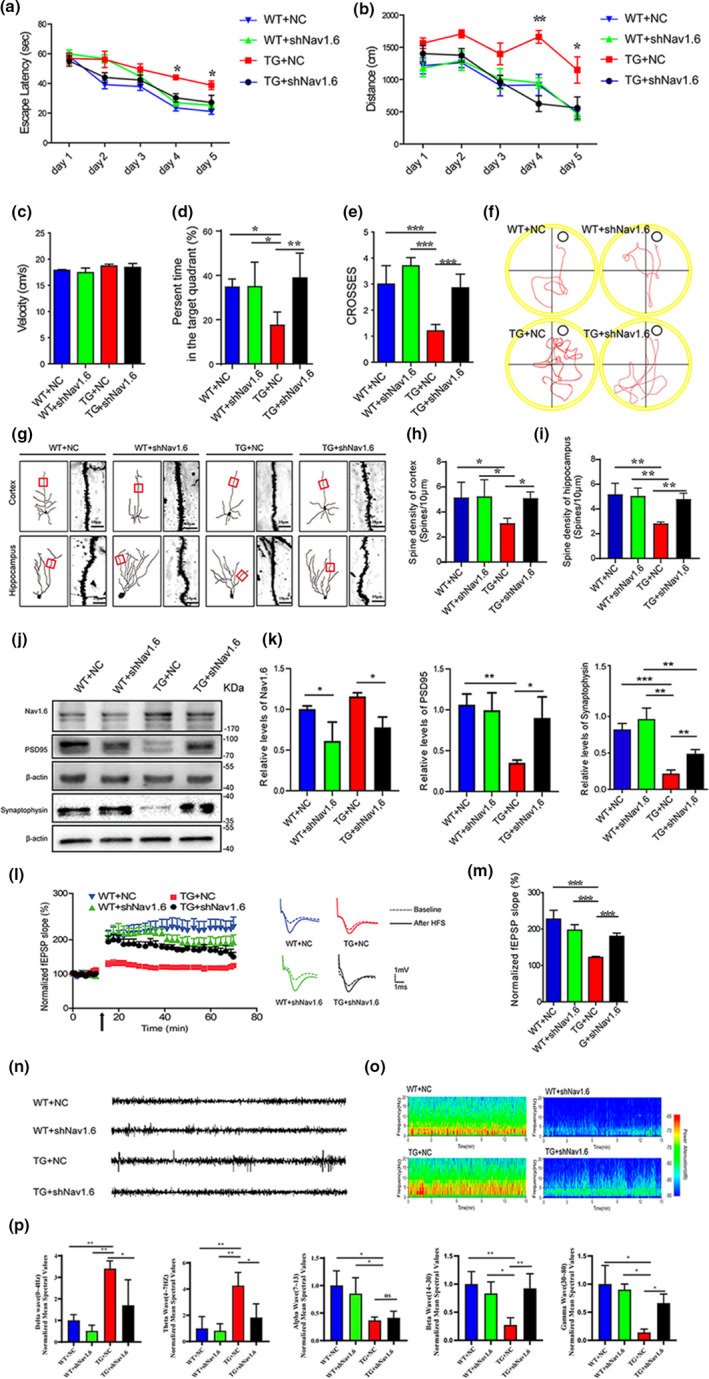
Nav1.6 knockdown attenuates cognitive deficits, ameliorates suppressed synaptic plasticity, and reduces hyperexcitability in APP/PS1 mice. (a–f) Acquisition of spatial learning in the different groups of mice after injecting with siNav1.6 and NC in the MWM with the hidden platform. (a) Escape latencies were measured for the APP/PS1 (TG) and wild‐type (WT) mice treated with either shNav1.6 or NC. (b) The average distance traveled by mice on the different trial days. (c) The swimming speed of the four groups of mice throughout the trial days. (d) The duration of stay in the target quadrant for four groups of mice. (e) The number of times that the four groups of mice swam across the target site after removal of the platform. (f) Representative images of the track visualization path that the mice would swim to find the platform. Data are presented as the mean ± SEM. *p < 0.05; **p < 0.01, ***p < 0.001. *n* = 7–9 mice/group. (g) Representative micrographs of dendritic spines in the four groups of mice (*n* = 5 mice/group; scale bar: 10 μm). (h, i) Bar graph showing the number of dendritic spines per unit micrometer (µm) in the cortex and hippocampus of four groups of mice. (j) Representative immunoblots and (k) densitometry analysis of synaptic protein (synaptophysin, PSD95) expression level in the four groups of mice. Here, synaptophysin and its corresponding β‐actin immunoblots were performed on different parts of PVDF membrane of the same gels (*n* = 8–10 mice/group). (l) Time course data on of the effects of HFS on the fEPSP initial slope. (m) Cumulative data showing the mean fEPSP slope 60 min post‐HFS (*n* = 5 mice/group, 3–4 slices per mice). (n) Representative traces of EEGs for the four groups of mice showing paroxysmal sinusoidal discharges in TG + NC and Nav1.6 knockdown reduced the paroxysmal sinusoidal discharges in TG + shNav1.6 mice. (o) Representative spectrograms of EEG for the four groups of mice. (p) Normalized differential band spectrum showed significant effects in delta, theta, beta, alpha, and gamma waves in the four groups of mice. Data are presented as the mean ± SEM. **p < 0.01; ***p < 0.001

Since the expression of Nav1.6 increased significantly in the brains of aged APP/PS1 mice, it was envisaged that Nav1.6 knockdown would alleviate the AD‐like symptoms. Therefore, adeno‐associated viruses (serotype 8, AAV8), which encode shRNA of Nav1.6 (Figure S1A,B; shNav1.6), were injected bilaterally into the hippocampus of 5‐month‐old APP/PS1 transgenic mice. AAV8 that encodes a scramble shRNA served as the control virus (NC). WT mice at a similar age were injected with either NC or shNav1.6 into the hippocampus. Three months postinjection, GFP labeling was detected by fluorescence imaging to confirm the location of injection (Figure S1C). Western blotting was used to confirm a marked reduction of Nav1.6 in brain by AAV‐shNav1.6 injection (Figure S1D,E). To examine whether knockdown of Nav1.6 rescues the cognitive deficits of APP /PS1 transgenic mice, Morris water maze (MWM) tests were performed 3 months after the injection of AAV8. As described previously, NC‐injected APP/PS1 transgenic mice (TG+NC) exhibited remarkable impairment of learning and memory in the task of locating the submerged escape platform, which was indicated by increased escape latencies (Figure 2a) and swimming distance (Figure 2b) in the probe trials, shorter duration of stay in the target quadrant (Figure 2d), and fewer times to swim across the platform zone in the reversal trials (Figure 2e,f). However, the APP/PS1 transgenic mice, which were injected with AAV encoding Nav1.6 shRNA(TG+shNav1.6), exhibited shorter escape latencies and swimming distances, more platform cross numbers, and presence time in the target quadrant than the TG+NC mice, also compared to the WT mice (Figure 2a–f). The above changes were not due to the swimming velocity, which remained identical across the four groups of mice (Figure 2c). Therefore, these results indicate that Nav1.6 knockdown rescues the cognitive deficits in APP/PS1 transgenic mice.

There is good consensus that impaired synaptic plasticity, including loss of dendritic spines and synapses, along with suppressed long‐term potentiation (LTP), is another feature of AD pathology. In order to examine whether knockdown of Nav1.6 attenuates synaptic plasticity impairment, we analyzed the density of dendritic spines, synaptic protein levels and LTP. Compared with the WT mice, TG+NC mice exhibited impaired synaptic plasticity as indicated by the reduction in the density of dendritic spines detected by Golgi staining (Figure 2g–i). The levels of synaptophysin (a specific marker for presynaptic terminals) and PSD95 (a membrane‐associated proteins in the postsynaptic density) were reduced (Figure 2j,k). In the LTP recording of the hippocampus, there was a significant dip observed in the field excitatory postsynaptic potential (fEPSP) slope (Figure 2l,m). Meanwhile, hippocampal knockdown of Nav1.6 via AAV enhanced the density of dendritic spines, synaptophysin and PSD95 expression, and LTP, to levels comparable to those of WT mice (Figure 2g–m). These results, taken together, indicate that Nav1.6 knockdown rescues the impaired synaptic plasticity in APP/PS1 transgenic mice.

EEG was recorded to observe the spontaneous and rhythmic electrical activity in each group of mice. Consistent with the previous report (Zhang et al., 2014), APP/PS1 mice exhibited neural hyperexcitability in the hippocampus as indicated by the increased frequency of epileptic spikes in EEGs. This was rescued by knockdown of Nav1.6 in the hippocampus of APP/PS1 mice (Figure 2n,o). Neural data recorded and analyzed from specific bandwidth were analyzed to know the effect of Nav1.6 knockdown on the various wave classifications. Power spectra of Delta and Theta waves were significantly increased in TG mice, whereas power spectra of alpha, beta, and gamma waves were significantly reduced in TG mice. Meanwhile, knockdown Nav1.6 can bring delta, theta, beta, and gamma waves associated with learning and memory back to normal levels (Figure 2p).

### 2.3 Knockdown of Nav1.6 inhibits the accumulation of Aβ and the cleavage of APP by β‐secretase via suppressing transcription of BACE1

**FIGURE 3:**
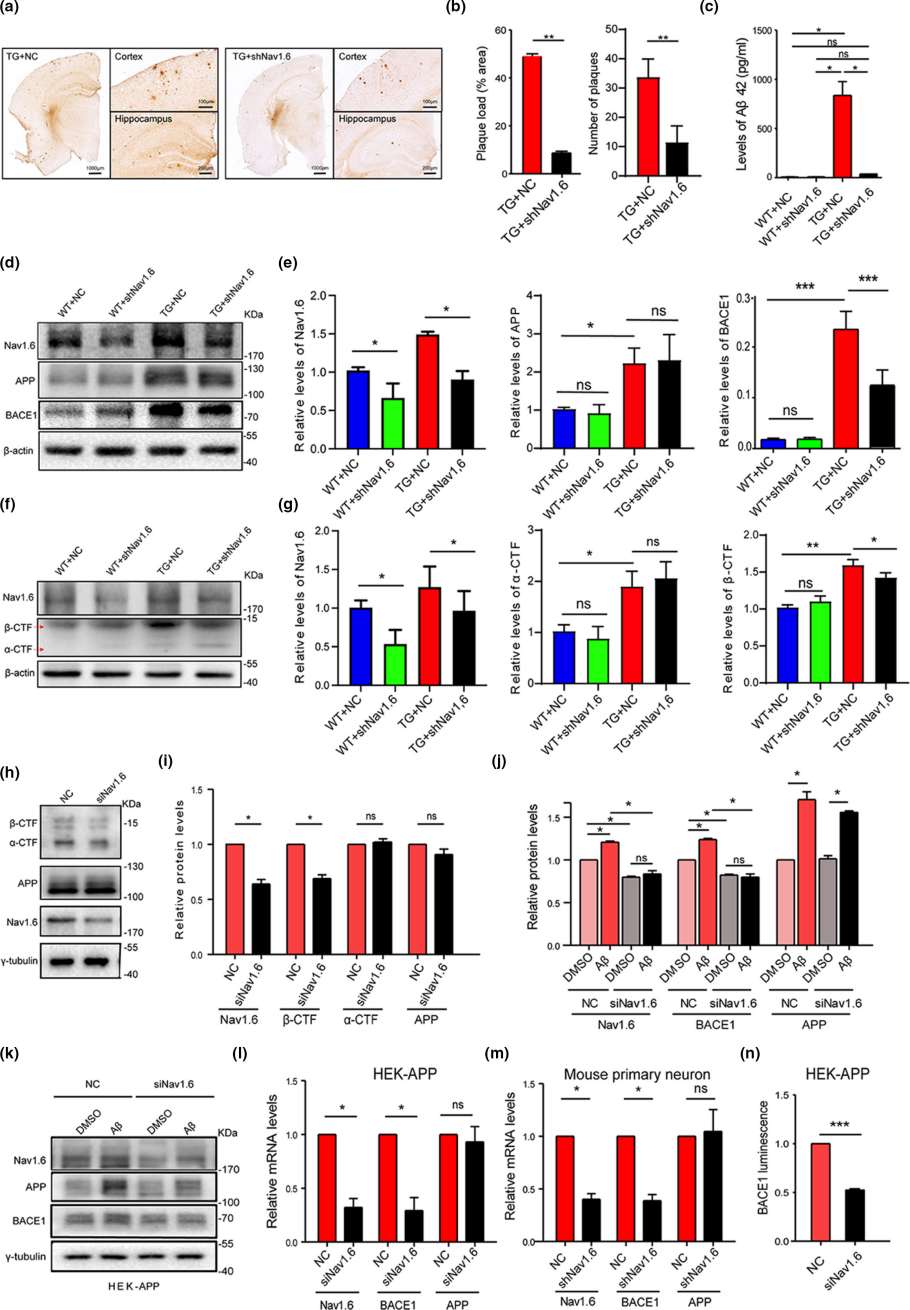
Nav1.6 knockdown inhibits the accumulation of Aβ and the cleavage of APP by β‐secretase by suppressing transcription of BACE1. (a) Coronal sections of the hippocampus were immunohistochemically stained with an antibody against Aβ. (b) The size and number of Aβ plaques were quantified in APP/PS1 treated with siNav1.6 and NC (*n* = 5 mice/group, 3 slices per mice). (c) Protein levels of Aβ42 using ELISA in the four groups of mice (WT and APP/PS1 treated with siNav1.6 or NC); *n* = 5 mice/group. Representative immunoblots (d) and densitometry analysis (e) of Nav1.6, BACE1, and APP protein expression in the brain of mice (WT and APP/PS1 treated with siNav1.6 or NC), *n* = 5 mice/group. Representative immunoblots (f) and densitometry analysis (g) of Nav1.6, α‐CTF, and β‐CTF protein expression in mice brain (WT and APP/PS1 treated with siNav1.6 or NC), *n* = 5 mice/group. Representative immunoblots (h) and densitometry analysis (i) of Nav1.6, β‐CTF, α‐CTF, and APP in HEK‐APP cells after knockdown with siNav1.6. Here, Nav1.6, β‐CTF, α‐CTF, APP, and their corresponding γ‐tubulin immunoblots were performed on different parts of the PVDF membrane of different gels. γ‐tubulin was used as a loading control (*n* = 4–5 groups). Data are presented as mean ± SEM. *p < 0.05, and **p < 0.01. Representative immunoblot (k) and densitometry analysis (j) of BACE1, APP, and Nav1.6 in the HEK‐APP cell line after treatment with NC and siNav1.6. Relative mRNA expression level of Nav1.6, BACE1, and APP in the HEK‐APP cell line (l) and mouse primary neurons (m) after treatment with NC and siNav1.6/shNav1.6, respectively. The relative expression was normalized to β‐actin. (n) Luminescence density of BACE1 in HEK‐APP cell line using the fluorescein reporter gene detection system after treatment with NC and siNav1.6 (3–5 biological replicates). Here, BACE1 (in the animal model) was normalized to β‐actin, BACE1, APP, and Nav1.6 (in HEK‐APP cell line) were normalized to γ‐tubulin in the Western blots, whereas Nav1.6, BACE1, and APP were normalized to actin in the RT‐PCR. Data are presented as mean ± SEM. *p < 0.05, ***p < 0.001

We explored the mechanisms underlying the beneficial effects of Nav1.6 knockdown in synaptic plasticity and cognition. Aβ is one of the central initiators of AD pathogenesis. Recent studies indicate that neuronal hyperexcitability is involved in the accumulation of Aβ plaques, but the mechanisms remain unknown (Bero et al., 2011; Yamamoto et al., 2015; Yuan & Grutzendler, 2016). First, we examined whether the Nav1.6 knockdown would attenuate the accumulation of Aβ plaques. We used the Aβ antibody (6E10) to stain the hippocampal sections and analyze the densities of Aβ plaques. We found that Aβ plaque density was significantly decreased after knockdown of Nav1.6 as evidenced by the reduction in both the numbers and the size of Aβ plaques in brains of TG+shNav1.6 mice (Figure 3a,b). This result was confirmed by ELISA analysis, which demonstrated the decreased levels of Aβ42 in the hippocampus of TG+shNav1.6 (Figure 3c). We further explored whether Nav1.6 knockdown affects the production of Aβ by interfering with the cleavage of APP. APP is cleaved extracellularly by either α‐secretase or β‐secretase, generating a truncated transmembrane fragment called α‐ or β‐C‐terminal figments (α‐/β‐CTF), respectively. Thus, the levels of α‐/β‐CTF could reflect the cleavage of APP by either α‐secretase or β‐secretase. BACE1 is the β‐secretase in the brain. We thus examined whether knockdown of Nav1.6 would alter BACE1 expression. Consistent with previous reports, APP/PS1 transgenic mice exhibited elevated BACE1 and β‐CTF levels in the hippocampus. By contrast, TG+shNav1.6 mice showed lower BACE1 and β‐CTF levels than the transgenic mice injected with a scrambled shRNA (TG+NC), but the α‐CTF and full‐length APP expression had not changed between these two groups (Figure 3d–g). Following, we confirmed these results by carrying out in vitro experiments. HEK293 cells that stably expresses full‐length APP (HEK‐APP cells) were transfected with Nav1.6 shRNA and a scrambled shRNA (NC) (Figure S2). The results demonstrated that knockdown of Nav1.6 decreased the levels of β‐CTF in HEK‐APP cells. By contrast, α‐CTF and full‐length APP expression levels remained unchanged in Nav1.6 shRNA‐ and NC‐transfected cells (Figure 3h,i). These results are consistent with those of the animal experiment (Figure 3d–g). Thus, knockdown of Nav1.6 may decrease Aβ accumulation by suppressing the cleaving of APP by β‐secretase.

Knockdown of Nav1.6 decreased the expression of BACE1. By contrast, hippocampal injection of AAV encoding Nav1.6 shRNA failed to alter BACE1 levels in WT mice (Figure 3d,e), suggesting that Nav1.6 regulates BACE1 expression in a context‐dependent manner. As Aβ oligomers are known to upregulate both Nav1.6 and BACE1 expression (Picon‐Pages et al., 2020; Wang et al., 2016), we further examined whether upregulation of Nav1.6 contributes causally to the increased expression of BACE1 induced by Aβ oligomers. HEK‐APP cells were transfected with either Nav1.6 siRNA or a scrambled siRNA (NC) and treated with Aβ oligomers. The results showed that the levels of Nav1.6, BACE1, and APP were all increased upon Aβ oligomer treatment. However, the Aβ oligomer‐induced upregulation of all these proteins was reversed in the presence of Nav1.6 siRNA (Figure 3j,k). In order to further explore whether knockdown Nav1.6 affects the generation of BACE1, we detected changes in mRNA levels by using siRNA knocking down Nav1.6 in the HEK‐APP cell and the primary cultured neurons. Similarly, the transfection of Nav1.6 siRNA decreased the levels of BACE1 mRNA, rather than APP mRNA, in HEK‐APP cells (Figure 3l) and primary culture neurons (Figure 3m). Moreover, when a luciferase reporter driven by a BACE1 promoter was co‐transfected with Nav1.6 siRNA in HEK‐APP cells, the Nav1.6 siRNA‐transfected cells showed significantly lower luminescence density than NC‐transfected cells (Figure 3n). Collectively, these results indicate that knockdown of Nav1.6 suppresses BACE1 transcription.

### 2.4 Nav1.6 regulating BACE1 depends on its channel property

**FIGURE 4:**
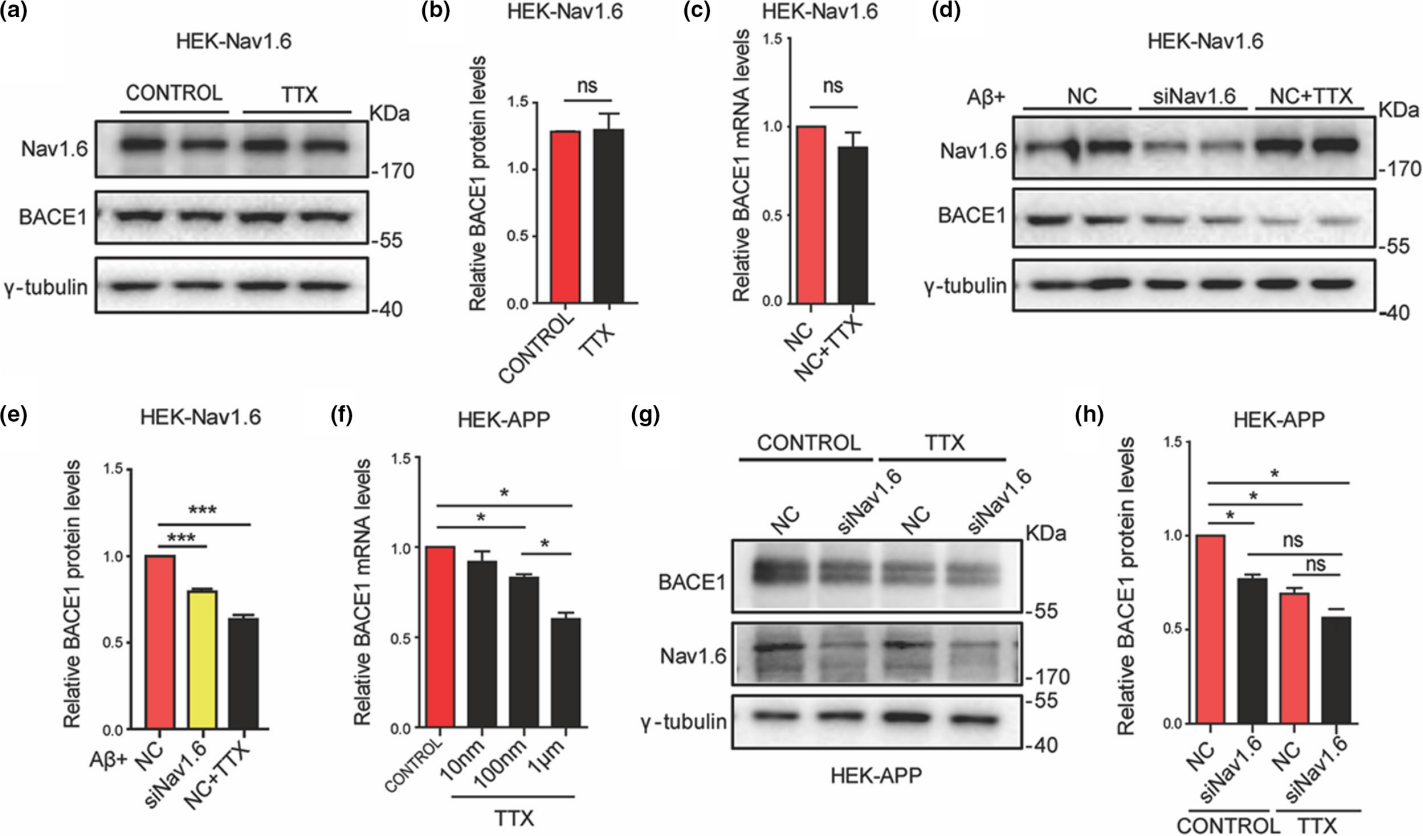
Nav1.6 regulates BACE1 expression dependent on its channel property. Representative immunoblots (a) and densitometry analysis (b) of BACE1 and Nav1.6 expression levels in the HEK cell line overexpressing Nav1.6 and treated with TTX (1 μM). (c) Relative mRNA expression level of BACE1 in the HEK cell line overexpressing Nav1.6 after treatment with TTX (1 μM). Representative immunoblots (d) and densitometry analysis (e) of BACE1 and Nav1.6 expression levels in the HEK cell line overexpressing Nav1.6 in the presence of induced Aβ oligomers. (f) Relative mRNA expression level of BACE1 in the HEK‐APP cell line after treatment with different concentrations of TTX. Representative immunoblots (g) and densitometry analysis (h) of BACE1 and Nav1.6 expression levels in the HEK‐APP cell line transfected with NC or siNav1.6 plasmids with or without TTX (1 μM). Here, BACE1, APP, and Nav1.6 were normalized to γ‐tubulin in the Western blot, whereas BACE1 was normalized to β‐actin in the RT‐PCR. Data are presented as mean ± SEM (*n* = 5 biological replicates). *p < 0.05, ***p < 0.001

Nav1.6, as a subtype of the sodium channel, participates in various biological processes via conducting the influx of Na^+^. Thus, we wondered whether Nav1.6 regulating BACE1 transcription is dependent on its role in conducting the influx of Na^+^. To investigate this possibility, HEK293 cells that stably expresses Nav1.6 (HEK‐Nav1.6 cells) were treated with 1 μM TTX, a nonselective sodium channel blocker, and BACE1 level was measured. The results showed that neither the protein nor mRNA levels of BACE1 was altered by the TTX treatment (Figure 4a–c), indicating knockdown of Nav1.6 suppressed BACE1 expression in a context‐dependent manner. The result was supported by Nav1.6 shRNA failing to reduce BACE1 levels in WT mice, though it did so in APP/PS1 mice (Figure 3d,e), indicating that Nav1.6 regulates BACE1 transcription in an Aβ‐dependent way. Furthermore, HEK‐Nav1.6 cells were either transfected with Nav1.6 siRNA or treated with TTX in the presence of Aβ oligomers in the culture medium. We observed that both Nav1.6 siRNA and TTX treatment decreased BACE1 levels in the presence of Aβ oligomers (Figure 4d,e). TTX altered BACE1 transcription in HEK‐APP cells, which express Nav1.6 endogenously and released a large amount of Aβ extracellularly, thus mimicking the condition associated with the presence of Aβ oligomers (Fan et al., 2013). This result showed that TTX inhibited BACE1 transcription in a dose‐dependent manner in HEK‐APP cells (Figure 4f). To further confirm the hypothesis that TTX suppresses BACE1 transcription via inhibiting Nav1.6, HEK‐APP cells were transfected with Nav1.6 siRNA and co‐treated with 1 μM TTX. In the presence of TTX, Nav1.6 siRNA failed to reduce BACE1 levels as it did in control cells (Figure 4g,h). Thus, these results indicate that the positive regulation of BACE1 transcription by Nav1.6 depends on its channel property, via regulating Na^+^ influx in the presence of Aβ.

### 2.5 Knockdown of Nav1.6 prevents Aβ oligomer‐induced upregulated BACE1 transcription through blocking abnormal calcium influx

**FIGURE 5:**
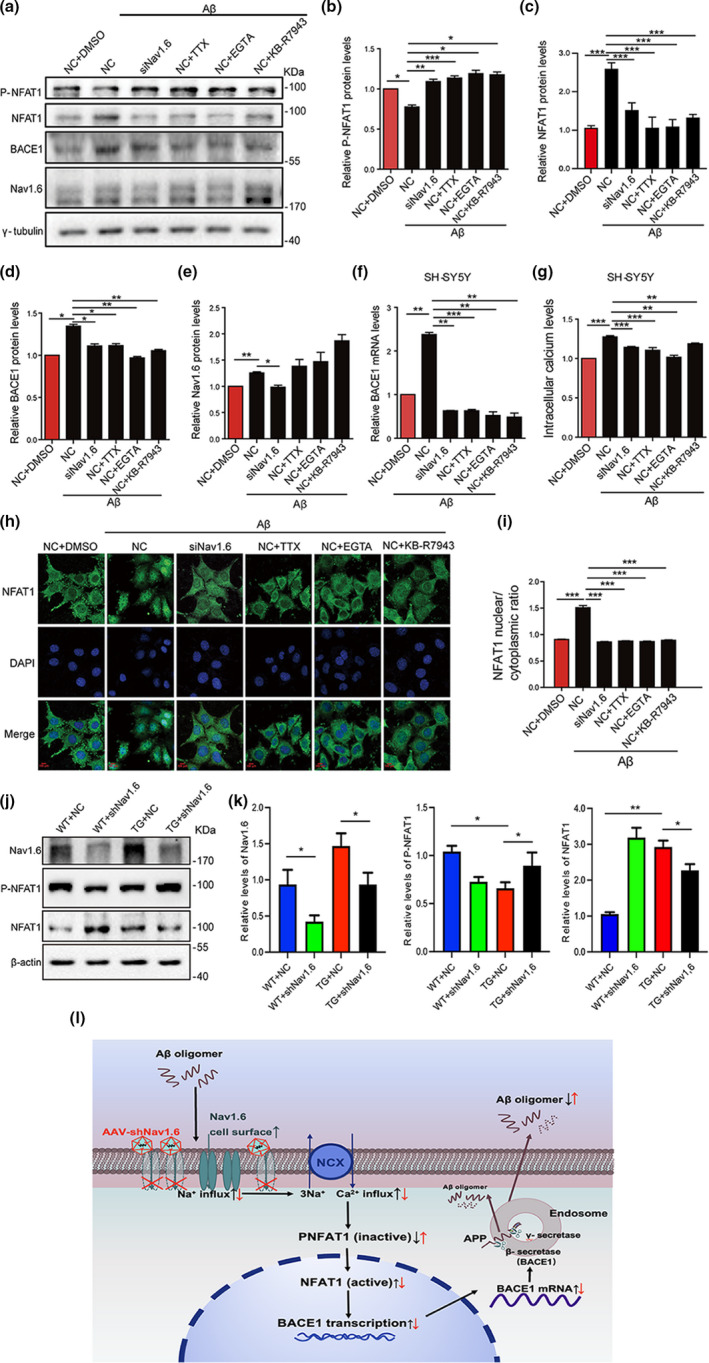
Aβ oligomer‐induced expression levels of BACE1 and inactive NFAT1 by interference with Nav1.6, TTX, EGTA, and KB‐R7943. Representative immunoblots (a) and densitometry analysis of P‐NFAT1 (inactive) (b), NFAT1 (active) (c), BACE1 (d), and Nav1.6 (e) expression levels in SH‐SY5Y cells after treatment with siNav1.6, 1 μM TTX, 0.5 mM EGTA, and 5 μM KB‐R7943 under induced Aβ oligomers condition. (f) Relative mRNA expression of RT‐qPCR showing the expression level of BACE1 in the SH‐SY5Y cells after treatment with siNav1.6, 1 μM TTX, 0.5 mM EGTA, and 5 μM KB‐R7943 under induced Aβ oligomers condition. (g) Intracellular calcium levels in SH‐SY5Y cell after treatment with NC, siNav1.6, 1 μM TTX, 0.5 mM EGTA, and 5 μM KB‐R7943 under induced Aβ oligomers condition. (h) SH‐SY5Y cells were either treated with 1 μM TTX, 0.5 mM EGTA, and 5 μM KB‐R7943 or transfected with Nav1.6 siRNA in the presence of Aβ oligomers and immunostained for NFAT1 and DAPI. Scale bar: 100 μm. (i) The ratio of nuclear NFAT1 to the cytoplasmic NFAT1. Here, P‐NFAT1, BACE1, and Nav1.6 were normalized to γ‐tubulin in the Western blot, whereas BACE1 was normalized to ACTIN in the RT‐PCR. Data are presented as mean ± SEM. *p < 0.05, **p < 0.01, **p < 0.001. Representative immunoblots (j) and densitometry analysis (k) of Nav1.6, P‐NFAT1, and NFAT1 protein expression in the brain of mice (WT and APP/PS1 treated with siNav1.6 or NC), *n* = 5 mice/group. (l) The proposed flowchart cycle depicting interference of Nav1.6’s effect on the progression of AD. Interference of Nav1.6 can reduce the Aβ oligomers‐dependent transcription of BACE1, which then relieves intracellular calcium overload by inhibiting sodium‐calcium reverse exchange channel and leads to an increase in non‐activated NFAT1 expression levels. The reduced transcription of BACE1, in turn, decreases Aβ production and slows the progression of AD

We have realized that from the above‐mentioned description that inhibition of Nav1.6 reduces BACE1 levels only in the presence of Aβ oligomers; otherwise, it fails. This result is consistent with our aforementioned finding that knockdown of Nav.16 does not affect learning and memory and synaptic plasticity in WT mice (Figure 2). Therefore, our results indicate that the accumulation of Nav1.6‐enhancing BACE1 transcription depends on Aβ oligomer‐mediated pathological process. Recently, researchers have focused on calcium dyshomeostasis based on the following evidence: 1) Nav1.6 enhances BACE1 transcription via regulating Na^+^ influx; 2) dysfunction of Nav1.6 in the injured axons induces Ca^2+^ influx via reversing the Na^+^‐Ca^2+^ exchanger (NCX) (Alrashdi et al., 2019; Craner et al., 2004; Omelchenko et al., 2019); and 3) elevated intracellular Ca^2+^ is an early pathological factor of AD and accelerates Aβ production, which is derived from the observation in AD patient brains (H. J. Cho et al., 2008; Demuro et al., 2010; Ishii et al., 2019). The elevated intracellular Ca^2+^ enhances BACE1 transcription by stimulating the nuclear translocation and activation of NFAT1, a transcription factor activated by calcineurin, and a calcium and calmodulin‐dependent phosphatase (H. J. Cho et al., 2008; Macian, 2005). Considering that Aβ increases the expression of Nav1.6 (Wang et al., 2016) (Figure 5a,e), we then further investigated whether Nav1.6 is involved or has any relationship with Aβ‐induced calcium influx. The intracellular Ca^2+^ concentrations in SH‐SY5Y cells were detected using the cell‐permeable fluorescent Ca^2+^ indicator Fluo‐4/AM kit. Our experiments found that the presence of Aβ oligomers increased the levels of intracellular Ca^2+^, which were rescued by either knockdown or inhibition of Nav1.6 by siRNA and TTX, respectively, or suppression of NCX by KB‐R7943 (an NCX inhibitor). Chelating Ca^2+^ by EGTA served as a positive control (Figure 5g). Interestingly, TTX, EGTA, and KB‐R7943 failed to alter the levels of Nav1.6, which were elevated by Aβ oligomers (Figure 5a,e). These results indicate that Aβ oligomers increase Ca^2+^ influx via reversed NCX. Upon activation, NFAT1 is dephosphorylated and translocated into the nuclei, where it functions as a transcriptional factor (H. J. Cho & Mook‐Jung, 2020; Macian, 2005). The levels of phosphorylated NFAT1 increased; however, nuclear NFAT1 decreased by either knockdown or inhibition of Nav1.6, chelating Ca2+ or suppressing NCX (Figure 5a,b,c,h,i), indicating that Aβ oligomers promote nuclear translocation and activation of NFAT1 via Nav1.6‐mediated Na^+^ influx and NCX‐induced Ca^2+^ influx. Consistent with the role of nuclear NFAT1 in BACE1 transcription, the upregulation of BACE1 mRNA and protein by Aβ oligomers was rescued by chelating Ca^2+^ or suppressing NCX as well (Figure 5a,d,f). Furthermore, experimenting with animals, we found that compared to the WT mice, the levels of p‐NFAT1 were reduced and NFAT1 was increased in TG+NC mice. Meanwhile, AAV‐assisted knockdown of Nav1.6 in the hippocampus enhanced the p‐NFAT1 expression and reduces the NFAT1 expression (Figure 5j,k). Therefore, these results suggest that Aβ oligomer enhances BACE1 transcription via Nav1.6‐mediated Na+influx and induces NCX transport reversal. Nav1.6 knockdown rescues Aβ oligomer‐induced Ca^2+^ influx, thus preventing BACE1 upregulation.

## 3 DISCUSSION

The results of this study indicate that Nav1.6, a subtype of sodium channel predominantly expressed in excitatory pyramidal neurons, is upregulated in APP/PS1 transgenic AD mice brains, which enhances Aβ generation via elevating transcription of BACE1, the key rate‐limiting enzyme in Aβ production. Knockdown of Nav1.6 with AAV attenuates Aβ accumulation and neuroinflammation also rescuing the impaired synaptic plasticity, and cognitive deficits. Our study presents a novel mechanism underlying neural hyperexcitability in AD pathogenesis and reveals the action of Nav1.6 as a novel target for AD therapy.

Neural network hyperexcitability is an early event in AD pathogenesis, which is even detected in the individuals at risk of developing dementia (Filippini et al., 2009) and in the patients with mild cognitive impairment considered to be a prodromal stage of AD (Dickerson et al., 2005). Thus, the appearance of network hyperexcitability in AD is suggested as a predictor of accelerated cognitive decline (Cretin et al., 2016; Vossel et al., 2016). The mechanisms underlying neural hyperexcitability in AD remain unclear. It has been reported that dysfunctional GABAergic transmission contributes to neural hyperexcitability in AD brains and dysfunction of SST^+^ interneurons and PV^+^ interneurons is associated with the cognitive deficits in AD (Schmid et al., 2016; Verret et al., 2012). Intriguingly, decreased levels of Nav1.1 (a subtype of sodium channels predominantly expressed in PV^+^ interneurons) have been observed on the surfaces of cells in the brains of AD patients and AD model mice (Verret et al., 2012). Transplantation of GABAergic interneurons rescues cognitive deficits of AD model mice without interfering with Aβ pathology (Lu et al., 2020; Martinez‐Losa et al., 2018; Tong et al., 2014), indicating that GABAergic dysfunction contributes to the cognitive deficits associated with AD independent of Aβ accumulation. As previously mentioned, we observed that Nav1.6, another subtype of the voltage‐dependent sodium channel, predominantly expressed in pyramidal neurons, was upregulated in the brains of AD model mice. Nav1.6 is predominantly localized at the initial gap segments of myelinated axons’ node of Ranvier, where it adapts to the high‐frequency stimulation of neurons, initiating, and propagating the action potential (O'Brien & Meisler, 2013). Moreover, Nav1.6 has an intrinsic ability to generate persistent and resurgent current, along with repetitive neuronal firing. Persistent sodium current, which is a subthreshold sodium current, makes the membrane depolarization longer to facilitate repetitive firings, and thus is important for regulating neuronal excitability (Patel et al., 2016). Soluble Aβ interferes with the function of Nav1.6 subunits directly and/or indirectly, leading to persistent sodium current amplitude increased (Ren et al., 2014), whereas the persistent sodium current partly accounts for the Aβ‐induced neuronal hyperexcitation (Ren et al., 2014; Wang et al., 2016). Riluzole (RLZ), an antagonist of persistent sodium current, suppresses persistent sodium current augmentation and the Aβ‐induced neuronal hyperexcitation (Ren et al., 2014). Therefore, the upregulation of Nav1.6 seems to contribute to neural hyperexcitability in AD brains. Consistent with this idea, the present study reveals that knockdown of Nav.16 decreased the frequency of epileptic spikes in the brains of APP/PS1 transgenic mice (Figure 2n). As observed in clinical studies involving EEG metric trend of AD (Gaubert et al., 2019), increased delta and theta power and decreased beta and gamma power bear considerable similarity with our results, which is consistent with our finding in APP/PS1 mice, whereas knockdown of Nav1.6 can bring delta, theta, beta, and gamma waves back to normal levels.

Suppression of neural hyperexcitability via distinct approaches such as transplantation of GABAergic interneurons, pharmacological manipulation, sensory deprivation, or chemogenetic inhibition attenuates cognitive deficits in AD (Bakker et al., 2012; Bero et al., 2011; Haberman et al., 2017; Lu et al., 2020; Martinez‐Losa et al., 2018; Sanchez et al., 2012; Tong et al., 2014; Yuan & Grutzendler, 2016; Zhang et al., 2014). These highlight early intervention of neural hyperexcitability is a promising therapeutic strategy for AD. The potential mechanisms of neural hyperexcitability contribute to cognitive deficits of AD include regulating neurotransmitter release, synaptic remodeling, gamma activity, or network stability (Kleen et al., 2013; Sanchez et al., 2012; Verret et al., 2012), which may be independent of Aβ accumulation (Lu et al., 2020; Sanchez et al., 2012; Tong et al., 2014). However, a close link between neural hyperexcitability and Aβ pathology has been explored in AD brains. The hyperactivity has been observed in the neurons in proximity to Aβ plaques in mouse models of AD (Busche et al., 2008, 2012), which chronically accelerates the accumulation of Aβ plaques (Yamamoto et al., 2015), whereas the suppression of neuronal hyperexcitability decreases the formation of Aβ plaques (Yuan & Grutzendler, 2016). These data implicate that neural activity is a critical driving force of Aβ accumulation. Accumulation of Aβ oligomers, in turn, increases neural excitability (Minkeviciene et al., 2009; Wang et al., 2016). Thus, a vicious cycle exists between Aβ accumulation and neural hyperexcitability.

Neural activity regulates Aβ release at presynaptic terminals and somato‐dendritic structure (Wei et al., 2010). In this context, it is worth noting that the accumulation of intraneuronal Aβ also leads to toxic effects on synaptic plasticity (Billings et al., 2005). Neural activity‐dependent Aβ release theory seems unable to explain the beneficial effects on learning and memory based only upon suppression of neural hyperexcitability, implying the existence of other possibilities. In this study, we report that Nav1.6, which plays a central role in initiation and propagation of action potential as well as neuronal excitability, increases its expression in the brains of AD model mice, and regulates BACE1 transcription. Knockdown of Nav1.6 in hippocampal neurons decreases BACE1‐mediated APP cleavage and subsequently Aβ generation, and rescues the deficits in learning and memory in AD model mice. TTX, a sodium channel blocker, also decreases BACE1 levels without altering Nav1.6 expression in cultured cells. In contrast, knockdown of Nav1. 6 fails to decrease BACE1 levels in the presence of TTX, suggesting that Nav1.6 regulates BACE1 transcription in a way dependent on Na^+^ currents. Therefore, our study provides a novel mechanism underlying neural activity in AD pathogenesis. This new proposed mechanism is consistent with the fact that neural hyperexcitability already exists in the very early phase of AD, highlighting that neural hyperexcitability acting as the initial factors impair synaptic plasticity and accelerates Aβ generation and accumulation in AD pathogenesis. Our results are also in accord with the finding that lamotrigine, a sodium channel blocker, reduces Aβ generation and accumulation in APP/PS1 transgenic mice (Zhang et al., 2014). They explain levetiracetam, which does not modulate neuronal VGSCs (Klitgaard, 2001), rescues the cognitive deficits of AD model mice without interfering with Aβ accumulation (Sanchez et al., 2012). Collectively, these data indicate neural hyperexcitability regulates Aβ production via upregulating Nav1.6.

Elevated intracellular calcium, which can be induced by Aβ accumulation, is an early hallmark of AD pathogenesis. Dysregulation of intracellular calcium homeostasis gives rise to Aβ production, the formation of Aβ plaques, Aβ‐induced toxicity, even neurodegeneration. Elevated intracellular calcium concentration increases BACE1 transcription via phosphorylating and translocating NFAT1 into the nucleus. NFAT is a transcription factor that can be activated by calcineurin, a calcium, and calmodulin‐dependent phosphatase (Macian, 2005). In the resting state of cell, NFAT is phosphorylated and present in the cytoplasm. When the intracellular calcium concentration is increased, calcineurin is activated and dephosphorylates NFAT. At this movement, NFAT1 is translocated into the nucleus, where it activates the process of BACE1 transcription (Cho et al., 2008). We have observed that Nav1.6 is involved in Aβ‐induced calcium influx. Either Nav1.6 knockdown or blocking of sodium channels with TTX prevents the Aβ‐induced calcium influx (Figure 5). Inhibiting of NCX also produces a similar effect. It is reported that NCX colocalizes with Nav1.6 in the injured axons (Craner et al., 2004). Aβ causes axonal injury and dysfunction. In addition, Aβ oligomers upregulate Nav1.6 expression resulting in the persistent sodium currents increase. Both cases would result in the accumulation of sodium ions (Nagai et al., 1989; Ciccone et al., 2019), reversed transportation of NCX, eventually leading to calcium influx. Elevated levels of intracellular calcium translocate NFAT1 to the nucleus and trigger BACE1 transcription (Figure 5l).

In view of the results from this and previous studies, it is important to seek alternative ways of regulating BACE1 expression. Knockdown of Nav1.6 can reduce the Aβ oligomers‐dependent transcription of BACE1, which can relieve intracellular calcium overload by inhibiting sodium‐calcium reverse exchange, leading to an increase in nonactivated NFAT1 expression levels. Reduced transcription of BACE1, in turn, decreases Aβ production, suppressing the progressive cycle and halting the pathophysiology of AD (Figure 5l). To the best of our knowledge, this is the first work that shows downregulation or inhibition of Nav1.6 could affect the Aβ production; therefore, Nav1.6 may become a novel therapeutic target to slow down the progression of AD.

## 4 EXPERIMENTAL METHODS AND MATERIALS

### 4.1 Experimental animals

Male APP/PS1 transgenic mice co‐expressing mutant human APP and PS1 with C57BL/6J background (Jackson Laboratory, 004462) were housed under specific pathogen‐free conditions (i.e., temperature 24℃, 12 h light/dark cycle, and relative humidity 45%–65% ad libitum). All experiments involving mice were approved and performed in accordance with the regulations of the Institutional Animal Care and the animal studies committee of Dalian Medical University (Ethics committee approval permit No. L2013011).

### 4.2 Antibodies and inhibitors

A rabbit polyclonal antibody against APP (1:1000) (A8717, Sigma), rabbit polyclonal anti‐Nav1.6 (1:200) (ASC‐009, Alomone Laboratories), rabbit polyclonal anti‐Nav1.2 (1:200) (ASC‐002, Alomone Laboratories), BACE1 (1:1000) (ab2077, Abcam), Phospho‐NFAT1 (1:500) (AF8011, Affinity Biosciences), NFAT1 (1:500) (DF7189, Affinity Biosciences), β‐actin (1:2000) (ab8227, Abcam), and γ‐tubulin (1:2000) (T6557; Sigma) were obtained from the respective commercial sources. TTX (1 µM, Sigma), EGTA (0.5 mM, sigma), Aβ1‐42 (5 µM, A‐1163–2, rPeptide), and KB‐R7943 (5 µM, MCE) were purchased.

### 4.3 siRNAs, plasmids, and virus

The sequences of siRNA for mouse Nav1.6 sense GCUGUCAGUCGUGAUGAUCTT, anti‐sense GAUCAUCACGACUGACAGCUU; negative control (NC) siRNA sense UUCUCCGAACGUGUCACGUTT, anti‐sense ACGUGACACGUUCGGAGAATT. The sequences of siRNA for human Nav1.6 sense GAGGGAUACCAGUGUAUGATT, anti‐sense UCAUACACUGGUAUCCCUCUG; NC sense UCUCCGAACGUGUCACGUTT, anti‐sense ACGUGACACGUUCGGAGAATT. Nav1.6 shRNA was constructed into pAKD. CMV‐bGlobin.eGFP.H1.shRNA vector was purchased from OBiO Biotechnology (Shanghai) Co., Ltd. The interference sequences of Nav1.6 were shRNA GCATGAAAGCAGGAAGAAATC and NC shRNA TTCTCCGAACGTGTCACGT. Nav1.6 shRNA plasmids were packaged into an adeno‐associated virus, serotype 8 (AAV8) by Heyuan Biotechnology, China.

### 4.4 Virus injection

NC and shNav1.6 AAV were injected into the hippocampus bilaterally (−2.1 mm anteroposterior from bregma, ±1.8 mm mediolateral from bregma, and 1.8 mm below the surface of the skull) of 5‐month‐old C57BL/6 mice and APP/PS1 transgenic mice using a stereotaxic apparatus. 1 µl of the virus suspension (2 × 10^12^ v.g./ml) was injected into each mouse hippocampus through a 10‐µl gauge needle at a rate of 0.2 µl/min. After injection, the needle was left in place for an additional 5 min before being slowly withdrawn from the site. Mice were recovered on a heating pad upon revival.

### 4.5 Cell surface biotinylation

Cell surface biotinylation was performed according to a protocol described previously (Liu et al., 2010). Briefly, samples obtained from the mouse cortex and hippocampus were isolated and incubated with 0.5 mg/ml sulfo‐NHS‐Biotin (Pierce) in ASCF bubbled with 95% O_2_ and 5% CO_2_ on ice for 30–40 min. The reaction was stopped using a quench buffer (Pierce). The tissues were then extracted in a lysis buffer containing 10 mM Tris‐HCl at pH 9.0, 150 mM NaCl, 0.5% Triton X‐100, 1% sodium deoxycholate, 0.5% SDS, 2 mM EDTA and protease inhibitor. Biotinylated proteins were captured by Streptavidin beads (Pierce) at 4°C overnight.

### 4.6 Western blot analysis

Western blotting was done according to our previously described protocol (Ntim et al., 2020). Briefly, to prepare whole extracts, frozen tissues (from the cortex and hippocampus) were homogenized in a lysis buffer (50 mM Tris‐HCl pH 7.5, 5 mM EDTA, 1% Triton X‐100, and protease inhibitor (Roche Diagnostics). After ultracentrifugation (150,000 *g*, 4°C, 45 min), the supernatants were collected and stored at −80°C for future use. All samples were subjected to a Bradford protein assay. Equal amounts of protein were separated on acrylamide gels and transferred onto polyvinyl difluoride (PVDF) membranes. Western blotting was performed under standard conditions, applying rabbit polyclonal antibodies against APP (1:1000), Nav1.6 (1:200), Nav1.2 (1:200), BACE1 (1:1000), phosphorylated nuclear factor 1 of activated T cells (P‐NFAT1) (1:500), β‐actin (1:2000), and mouse monoclonal antibodies against γ‐tubulin (1:2000) at 4°C overnight. The latter antibody was used for loading normalization. Either anti‐mouse or anti‐rabbit peroxidase‐conjugated secondary antibodies were applied at 1:5000 for 1hr at room temperature and blots were visualized with an ECL detection kit (Amersham). Images obtained were quantified using the ImageJ software.

### 4.7 Cell culture, transfection, and drug treatments

Mouse primary cortical cultures were prepared from brains of P0 (postnatal day 0–1) C57BL/6J mice or APP/PS1 mice. Cells were maintained in a 37°C incubator with 5% CO_2_ supply for 7 days for the quantitative real‐time PCR (qPCR) assay. Human SH‐SY5Y neuroblastoma cells were also maintained in Dulbecco's modified Eagle's medium (DMEM; HyClone) supplemented with 10% fetal bovine serum (FBS; HyClone) and 1% penicillin/streptomycin antibiotic mixture at 37°C in a humid atmosphere of 5% CO_2_. Cells were treated with aggregated Aβ1–42 (5 µM, rPeptide) with or without tetrodotoxin (TTX, 1 µM, Sigma), ethylene glycol tetraacetic acid (EGTA, 0.5 mM, Sigma), or 2‐(2‐(4‐(4‐nitrobenzyloxy) phenyl ethyl isothiourea methanesulfonate (KB‐R7943, 5 µM, MCE) for 48 h. Aggregated Aβ1–42 was obtained by incubating at 37℃, 220 rpm for 48h as previously described (Jones et al., 2013). HEK‐APP cells and SH‐SY5Y cells were transfected by Lipofectamine 3000 (Thermo Fisher Scientific) according to the manufacturer's protocol. For the transfection of mouse primary neurons, lentivirus was added to the media.

### 4.8 Immunofluorescence Staining and Immunohistochemistry

Cells were fixed with 4% paraformaldehyde for 15 min, permeabilized with 0.3% Triton X‐100 for 5 min, and incubated with 10% bovine serum albumin (BSA) for 60 min at room temperature to block non‐specific binding. Without washing, the diluted primary antibodies (NFAT1, Affinity Biosciences) were added and incubated at 4℃ overnight. After three washes with PBS, cells were incubated with the corresponding secondary antibodies at room temperature for 1h. Images were acquired from a Laser confocal microscope (ZEISS, LSM 700). For immunohistochemistry, the mouse brain was sliced into brain slices with a continuous 10 μm coronal slice using a cryostat (Leica CM 1850, Leica Microsystems AG, Wetzlar, Germany), and sections were incubated with rabbit anti‐Aβ (1:500; 6E10, Biolegend) in 5% normal goat serum and 0.05% Triton X‐100 overnight at 4°C. The next day, sections were stained with 0.05% 3, 3′‐diaminobenzidine (DAB). The entire image was captured using a Pannoramic MIDI (3D Histech, Hungary) equipped with a GS3‐U3‐51S5M C camera (FLIR, Canada), Lumencor SOLA (Beaverton, OR) and Semrock filter (Rochester, NY). Quantified Aβ plaque load by divided the areas of Aβ plaque by the total area of the corresponding site. The image was analyzed with Image‐ProPlus 6.0 image analysis software (Media Cybernetics, MD, USA).

### 4.9 Real‐time quantitative polymerase chain reaction

Total cellular RNA was extracted using Trizol Reagent (Sigma‐Aldrich). An equal amount of the first‐strand cDNAs was synthesized with the FastQuant RT Kit (Tiangen Biotech, Beijing, China). PCRs were performed with Taq DNA polymerase (Takara, Dalian, China). The following primers were used.

**Table:** 

Mouse *Nav1*.*6*:	F: CTCCAAGAAGCCACAGAAGC
	R: ATGGAGAGGATGACCACCAC
Mouse *BACE1*:	F: GGAACCCATCTCGGCATCC
	R: TCCGATTCCTCGTCGGTCTC
Mouse APP:	F: TCCGAGAGGTGTGCTCTGAA
	R: CCACATCCGCCGTAAAAGAATG
Mouse *GAPDH*:	F: TCCACCACCCTGTTGCTGTAG
	R: GACCACAGTCCATGACATCACT
Human *Nav1*.*6*:	F: CCAAACTAAAGGTGCACGCC
	R: TGGAAGTCACCATTCCGGTG
Human *BACE1*:	F: ACCAACCTTCGTTTGCCCAA
	R: TCTCCTAGCCAGAAACCATCAG
Human APP:	F: CAAGCAGTGCAAGACCCATC
	R: AGAAGGGCATCACTTACAAACTC
Human *ACTIN*:	F: CATGTACGTTGCTATCCAGGC
	R: CTCCTTAATGTCACGCACGAT

### 4.10 Relative luciferase activity measures

HEK‐APP cells were seeded into a 24‐well plate and co‐transfected with PGL3‐hbace1‐luciferase, PGL4.7‐renilla, together with siNav1.6 or NC (as control) siRNA with 1 µl (20 nM)/ siRNA/ well. Cells were harvested using Glo Lysis Buffer (Promega) 48 h after transfection and analyzed using the Dual‐Luciferase^®^ Reporter Assay System (Promega). Luciferase activity was detected with a luminometer. Luciferase activities of firefly (LAF) and luciferase activities of renilla (LAR) were detected through a Microplate Reader (TECAN infinite M200 Pro).

### 4.11 Morris water maze

The Morris water maze test was performed as described by Morris (1984). Briefly, mice were taken through 4 trials per day. A different starting position was used in each trial. The duration of each trial was 90 s. Escape latencies (time spent swimming from the start point to the target) and path length (the distance from the start point to the platform) before reaching the platform were recorded for 5 consecutive days. The escape latency of mice at the first training day was normalized to 1.0. The relative escape latencies in the following training days were analyzed (escape latency in the following day/escape latency on the first day) and interpreted as a learning trend. For the probe trials, the platform was removed after the last trial of the acquisition period. The mice were tested 24 h later to assess memory consolidation. The time spent in the target quadrant within 60 s was recorded. The latency to the first target site was measured, and the numbers of platform‐site crossovers were recorded.

### 4.12 Analysis of spines

Brain sections were stained with the FD Rapid Golgi Stain kit (FD Neurotechnologies, Germantown, MD, USA). Images of stained spines were acquired from the LEIDA DM4000B fluorescence microscope (Leica) using a 40 oil‐immersion objective. Spines were estimated from the hippocampus. The spine density was calculated on dendrites with lengths of 10 µm. The density of the spines was quantified from about 100 randomly selected dendritic segments per slide (3 slides per mouse with 5 mice per group). The estimation was done by an independent researcher blinded to the groupings of the study.

### 4.13 Electroencephalograph recordings

Electroencephalogram (EEG) was performed to record the role of Nav1.6 in the electrical activity of mice brains. The method of electrode implantation and EEG recording has been modified according to literature. Electrode implantation was done by fixing the mice on stereotaxic apparatus (David Kopf Instruments, Model 902) and fitted with a ventilation mask that provided 2.5% of volatile isoflurane during the entire operation. Two screw electrodes (diameter 1.0 mm and length 2.0 mm) were fixed on both frontal bones with dental cement at AP 2.0 mm, ML ± 1.5 mm from bregma. One parietal screw electrode was implanted on the occipital bone above the cerebellum and was used as ground and reference electrodes. Postoperative recovery period for the mice was at least 7 days. All EEG recordings were carried out on mice moving freely in the test cage after recovery. The EEG activity in each mouse was monitored daily for up to 3 days with Powerlab 8/35 software (version 7, LabChart). EEG was recorded in a wide frequency band (0.01–40,000 Hz) and amplified 1000–2000 times.

### 4.14 Recording of long‐term potentiation

Recording of long‐term potentiation (LTP) was recorded in the hippocampus of C57BL/6 and APP/PS1 mice, which had been injected with NC or shNav1.6 AAV 3 months later. Mice were decapitated under isoflurane with pentobarbital, and the brain was quickly removed and placed in ice‐cold oxygenated artificial cerebrospinal fluid (aCSF) comprised of 185 mM sucrose, 2.5 mM KCl, 4 mM MgSO_4_·7H_2_O, 0.5 mM CaCl_2_·2H_2_O, 1.2 mM NaH_2_PO_4_, 26 mM NaHCO_3_, and 25 mM D‐glucose (pH 7.4). Coronal hippocampal slices (300 μm) were prepared from the resected brains of mice using a vibratome (Leica VT1200S). The slices were maintained at room temperature in artificial cerebrospinal fluid (aCSF) comprised of 124 mM NaCl, 3.3 mM KCl, 1.2 mM MgSO_4_·7H_2_O, 2.5 mM CaCl_2_·2H_2_O, 1.2 mM NaH_2_PO_4_, 26 mM NaHCO_3_, and 10 mM D‐glucose (pH 7.4) bubbled with 95% O_2_ and 5% CO_2_ for at least 60 min before transfer to a submersion recording chamber, which was continually perfused with oxygenated aCSF with GABA antagonists picrotoxin 100um/L at the rate of 1 ml/min. The field excitatory postsynaptic potentials (fEPSPs) in CA1 neurons were recorded by stimulating CA3 neurons. Long‐term potentiation (LTP) was induced by applying high‐frequency stimulation (HFS) (four 100 Hz and 1 s trains delivered 20 s apart). The LTP magnitude was quantified as the percentage change in the fEPSP slope (40%) taken during the 60‐min interval after LTP induction. The electrophysiological data were acquired with an Axon multiclamp 700 B amplifier, filtered at 0.1–5 kHz, and digitized at 10 kHz, and the slope and peak amplitude of fEPSP were measured and analyzed offline using pClamp10.3 software (Molecular Devices Corp, USA).

### 4.15 Analysis of the levels of Aβ

The hippocampus was homogenized in 400 μl TBS (20 mM Tris, 137 mM NaCl, pH 7.6) containing protease inhibitors (Roche, Germany). Homogenates were spun at 350,000 *g* for 30 min at 4°C. Then, the pellet was resuspended in 400 μl 5 M guanidine (50 mM Tris‐HCl, PH 8.0) with protease inhibitors, homogenized for 3–4 h at room temperature, and centrifuged at 15,000 *g* for 10 min at 4°C. The levels of Aβ were analyzed with human Aβ42 (KHB3441, Invitrogen, USA) ELISA kits according to the manufacturer's instructions.

### 4.16 Detection of intracellular calcium levels

Cells were treated with aggregated Aβ1–42 (5 µM, rPeptide) with or without TTX (1 µM, Sigma), EGTA (0.5 mM, Sigma), or KB‐R7943 (5 µM, MCE) when transfected with NC or siNav1.6 siRNA for 48h. Then, cells were incubated with the cell‐permeable fluorescent Ca indicator Fluo‐4/AM (2 µM, 37°C) for 30 min. Before measuring fluorescence, the cells were washed in an indicator‐free medium (containing an anion transport inhibitor, if applicable) to remove any dye nonspecifically associated with the cell surface, then incubated for an additional 30 min to allow complete de‐esterification of intracellular AM esters. The fluorescence intensity was detected with a microplate reader (TECAN infinite M200 Pro) using the appropriate wavelength settings (excitation at 485 nm, emission at 520 nm).

### 4.17 Statistical analysis

All statistical analyses were performed using SPSS 19.0. Data are expressed as mean ± SEM. Data from multiple groups were analyzed by one‐way analysis of variance (ANOVA). Repeated‐measures ANOVA was used to analyze the data from the Morris water maze. An independent sample *t*‐test was used for comparison between the two groups. A *p* value <0.05 was regarded as statistically significant in all analyses (*p < 0.05; **p < 0.01; ***p < 0.001).

## CONFLICT OF INTEREST

The authors declare that they have no competing interest in this publication.

## AUTHOR CONTRIBUTIONS

S. Li and Q‐H. Ma contributed to the conception and design of the project. D‐J Yuan, G Yang, W Wu, Q‐F Li, M. Ntim, C‐Y Jiang, X‐Y Na, Y‐Z Wang, D‐D Zhu, J‐C Liu, Y Zhang, K Supratik, and ZC Xiao contributed to the conduct of the experiments and analysis of data. D‐J Yuan and S. Li wrote the manuscript. M. Ntim helped with manuscript revision and proofreading. All authors read and approved the final version of the manuscript.

## Supporting information

Figures S1 and S2

## Data Availability

The data that support the findings of this study will be available from the corresponding author upon reasonable request.
