# Supplementary material for: Reducing Nav1.6 expression attenuates the pathogenesis of Alzheimer's disease by suppressing BACE1 transcription
Source: Aging Cell. 2022 Mar 30;21(5):e13593. doi: 10.1111/acel.13593 (PMC9124306; doi:10.1111/acel.13593)
Supplement: Supplementary file 1 — Figures S1 and S2 [file ACEL-21-e13593-s001.docx]

**Supplementary figure 1**


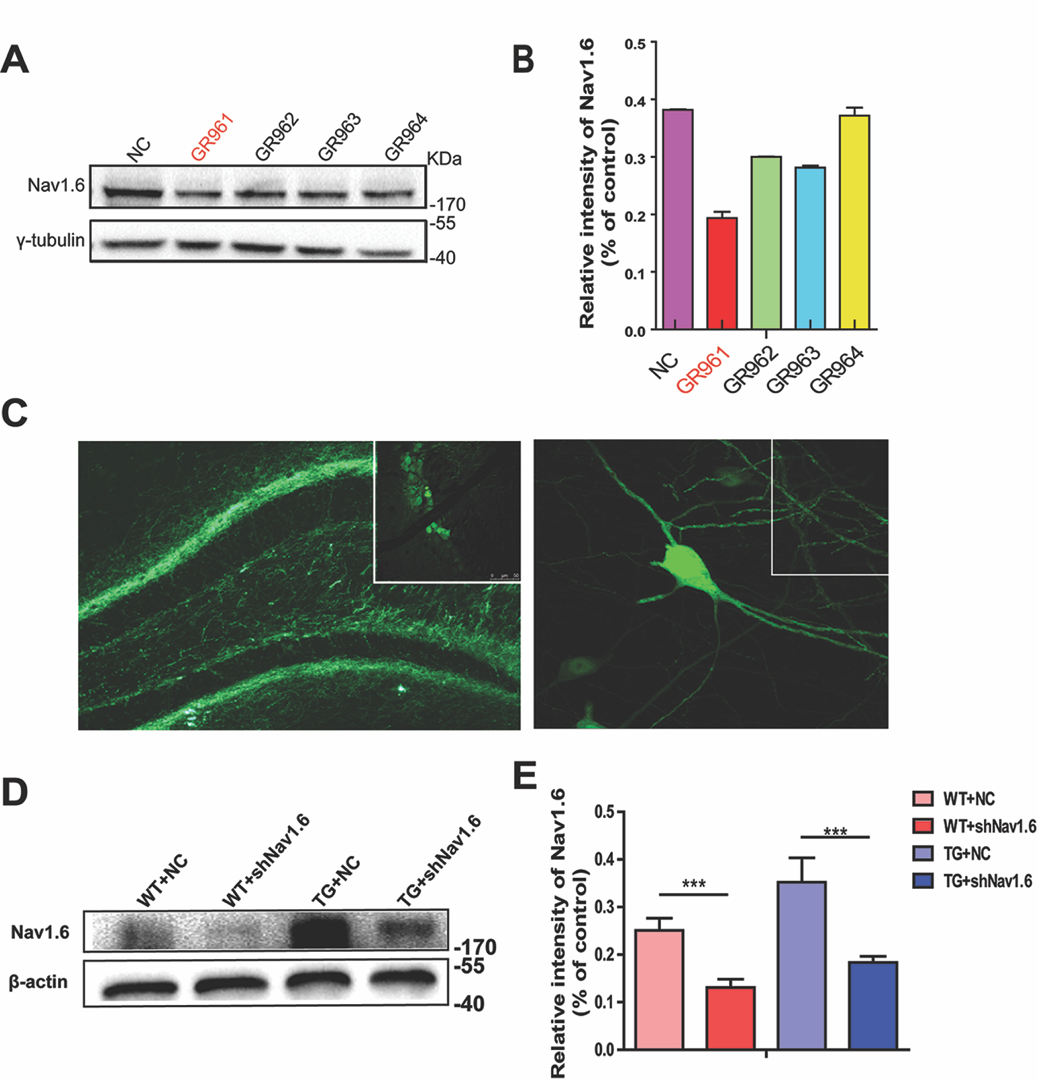


**Evaluation of the infection efficiency after injection/treatment of AAV expressing in reducing Nav1.6.** Representative immunoblots (A) and densitometry analysis (B) of Nav1.6 in samples after treatment with different combinations of AAV. (C) Fluorescence image of AAV-mediated GFP expression at the target area. Representative immunoblots (D) and densitometry analysis (E) of Nav1.6 in the groups after treatment with the selected shNav1.6. Data are presented as mean ± SEM. **** represent p<0.001*.

**Supplementary figure 2**


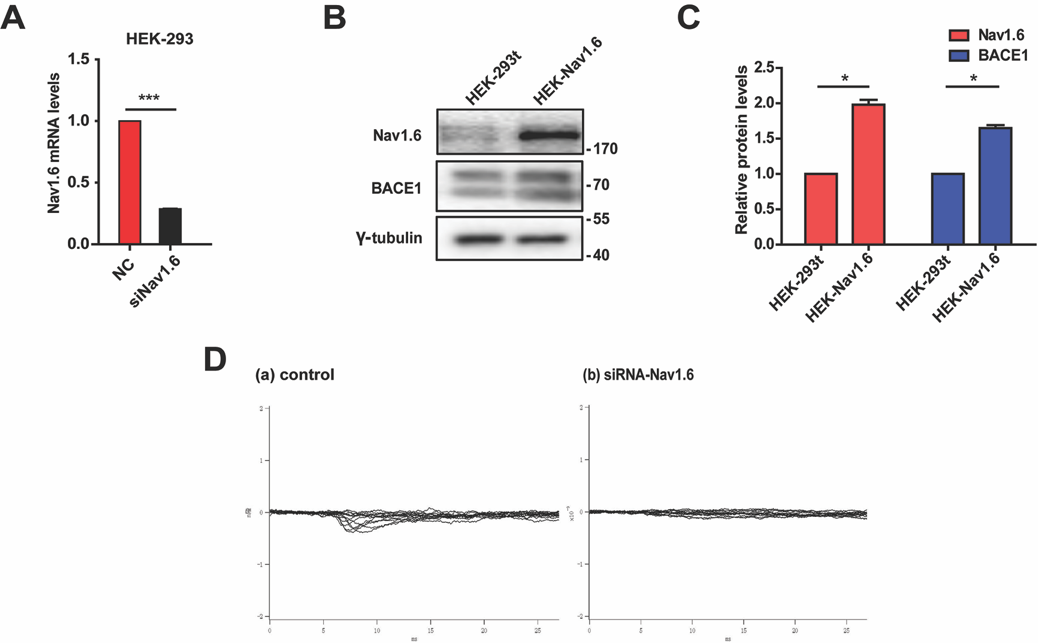


**Evaluating the efficacy of the shNav1.6 and siRNA-Nav1.6 in reducing Nav1.6 and Na^+^ current.** (A)Relative mRNA expression of Nav1.6 in HEK-293 cells after treatment with selected siNav1.6. Here, Nav1.6 was normalized to γ-tubulin in the western blots and normalized to β-actin in the RT-PCR.Representative immunoblots (B) and densitometry analysis (C) of Nav1.6 and BACE1 in HEK-293t and HEK-Nav1.6. (D) Representative traces of Na^+^ current that was recorded in the control versus cells treated with siRNA-Nav1.6. Here, Nav1.6 was normalized to β-actin in the animal model, whereas BACE1 and Nav1.6 were normalized to γ-tubulin in cells in the western blot. Data are presented as mean ± SEM. * *represents p < 0.05,* **** represent p<0.001*.
